# ZFP14 Regulates Cancer Cell Growth and Migration by Modulating p53 Protein Stability as Part of the MDM2 E3 Ubiquitin Ligase Complex

**DOI:** 10.3390/cancers14215226

**Published:** 2022-10-25

**Authors:** Shakur Mohibi, Xinbin Chen, Jin Zhang

**Affiliations:** Comparative Oncology Laboratory, Schools of Veterinary Medicine and Medicine, University of California, Davis, CA 95616, USA

**Keywords:** ZFP14, p53, Mdm2, protein stability, cancer cell growth

## Abstract

**Simple Summary:**

Cancer is among the leading causes of deaths in the US and worldwide. Although extensive research has led to a decline in the overall cancer deaths in the US since the early 1990s, cancer still remains a major cause of mortality in the US. The tumor suppressor *p53* is the most frequently mutated gene in all human cancers, accounting for a loss of function in more than 50% of all human cancers. Even after years of extensive research on p53, most of the pathways regulating the p53 family network are yet to be elucidated. Here, we identified a mutual regulation between p53 and ZFP14, a member of the largest family of transcription factors in humans, whose functions have been unknown till now. We showed that p53 can increase the amount of ZFP14 when cells are under stress and that ZFP14, in turn, negatively affects the p53 protein levels forming a feedback loop. We also showed that ZFP14 carries this out as part of a complex containing the major p53 negative regulator, MDM2. Moreover, the removal of ZFP14 from cancer cells reduces their tumorigenic properties in a manner dependent on p53 levels. Our findings reveal that ZFP14 might play an important role in tumor suppression via p53.

**Abstract:**

Multi-zinc finger proteins that contain a KRAB domain are part of the biggest family of transcription factors in mammals. However, the physiological or pathological functions for the majority of them are unknown. Here, we showed that *ZFP14* (also known as *ZNF531*) is a p53 target gene that can be induced upon genotoxic stress in a p53-dependent manner. To determine the function of ZFP14 in mouse and human cancer cell lines, we generated multiple cell lines where *ZFP14* was knocked out. We showed that *ZFP14-KO* inhibits cancer cell growth and migration. We also showed that, as a target of p53, ZFP14, in turn, represses p53 expression and that the knockdown of *p53* restores the potential of *ZFP14-KO* cells to proliferate and migrate. Mechanistically, we found that ZFP14 modulates p53 protein stability by increasing its ubiquitination via associating with and possibly enhancing MDM2/p53 complex integrity through its zinc finger domains. Our findings suggest that the reciprocal regulation of p53 and ZFP14 represents a novel p53-ZFP14 regulatory loop and that ZFP14 plays a role in p53-dependent tumor suppression.

## 1. Introduction

The KRAB-ZFPs (multi-zinc finger proteins containing Kruppel-associated box) are part of the largest family of transcription factors in vertebrates, especially in mammals [1,2]. Although largely considered as DNA-binding transcriptional repressors, they can also activate transcription, as well as bind to RNA and proteins to execute their key functions [2,3,4]. The N-terminal KRAB domain generally facilitates the recruitment of repressive complexes, whereas the signature residues in each of their zinc fingers aid in the recognition of specific nucleotides in DNA/RNA or specific amino-acids in proteins [3,5,6]. Despite their abundance and several large-scale studies showing their contribution to the evolution of gene-regulatory networks, their role in physiology and various diseases, including cancer, remains relatively rare [2,7,8].

The tumor suppressor *p53* is the most commonly mutated gene in human cancers. Upon various cellular stresses, p53 suppresses oncogenic traits by regulating various transcriptional networks that induce cell growth inhibition or cell-death pathways [9,10]. Although p53 is one of the most studied proteins, the pathways that mediate p53-dependent tumor suppression are not completely understood [9,10]. p53 is known to expand its transcriptional network by activating several transcription factors, including multi-zinc finger proteins, such as ZNF750, ZNF185 and Zfp871 [11,12,13]. Moreover, ZNF420, ZNF475 and ZNF568 directly interact with p53 and regulate the transcription of a small subset of p53-target genes [14,15]. On the other hand, several ZFPs, such as ZNF307, Zfp871 and Zfp148, regulate p53 protein stability by diverse mechanisms [12,16,17,18,19]. Thus, identifying more zinc finger proteins from this largely understudied family as regulators of p53-dependent tumor suppression will provide more insights into the p53 pathway.

Here, we established the KRAB zinc finger protein ZFP14 as a p53 target gene that is induced upon genotoxic stress in a p53-dependent manner. Moreover, *ZFP14-KO* cancer cells showed a decrease in cell growth and migration, which was a result of the negative regulation of the p53 protein by ZFP14. Mechanistically, we found that ZFP14 modulates p53 protein stability by associating with the MDM2/p53 complex and increasing p53-poly-ubiquitination and degradation. Overall, our findings suggest that ZFP14 is an important regulator of p53 during tumorigenesis.

## 2. Materials and Methods

### 2.1. Cell Culture, Cell Line Generation and Reagents

The mouse C2C12 cell and the human HCT116, MCF7 and RKO cells were purchased from American Type Culture Collection (ATCC) and used within 2 months of thawing or below passage 20. Dulbecco’s modified Eagle’s medium (DMEM) (Invitrogen) supplemented with 10% fetal bovine serum (FBS) (Hyclone, Logan, UT, USA) was used to culture all of the cell lines. Generation of *ZFP14-KO* HCT116 and MCF-7 cell lines was achieved by transfecting two pSpCas9(BB)-2A-Puro vectors (Addgene plasmid#62988) expressing single guide RNA (sgRNA) to remove *ZFP14* exon 3 and create a frame shift deletion. The two guide RNAs would create an approximately 287 base pair deletion leading to a frame shift from amino acid 4 onwards. Puromycin was used to select the transfected cells, and the clones obtained were genotyped using the primers: sense, 5′-CAA AAG GCT TGC GTG CAC TA-3′, and antisense, 5′-GGC CTA GTT CCA ACC TGA CAA-3′. The bands obtained from the genotyping were sequenced to confirm the deletion of the entire exon 3. The clones were further tested to show loss of *ZFP14* mRNA by RT-PCR. The clones that did not show the deletion were sequenced and used as isogenic controls if the sequence in the targeted region was not mutated or deleted.

### 2.2. Plasmids and siRNAs

Plasmids for pcDNA3-p53 and pcDNA3-2x-FLAG-Ubiquitin have been described previously [20,21]. The pcDNA3-(mouse)Zfp14-FLAG ORF clone (Cat#OMu07658D) was purchased from GenScript (Piscataway, NJ, USA). The cDNA clone for human *ZFP14* (cloneid: 9053225) was obtained from Dharmacon (Lafayette, CO, USA). The human *ZFP14* ORF was amplified from the cDNA clone using the primers: forward (containing HA-tag)-5′ AGT CAA GCT TAT GTA CCC ATA CGA TGT TCC AGA TTA CGC TGC CCA TGG TTC AGT GAC ATT-3′; and reverse–5′-AGT CCT CGA GTT AAA TTC CAT TAT GAA TCT-3′. The resulting PCR amplicon was digested with HindIII-XhoI and cloned in pcDNA3 and pcDNA4 vectors digested at the same sites. The clones obtained were sequence verified.

pSpCas9(BB)-2A-Puro vectors containing *ZFP14* specific sgRNAs were generated based on the previously described protocol [22]. The oligonucleotides used for cloning sgZFP14-1 are sense, 5′-CAC CGG ATA GCA TGC AAA CAG CAT T-3′, and antisense, 5′-AAA CAA TGC TGT TTG CAT GCT ATC C-3′; for sgZFP14-2, are sense, 5′-CAC CGC TTT CTC TGC TGT CAA TTT C-3′, and antisense, 5′-AAA CGA AAT TGA CAG CAG AGA AAG C-3′.

All of the small interfering RNAs (siRNAs) used in this study were purchased from Dharmacon RNA Technologies (Lafayette, CO, USA) and are listed in Supplementary Table S1.

### 2.3. Western Blot and IP-Western Blot Analysis

Western blotting was performed as previously described [23]. Briefly, 7–11% SDS-polyacrylamide gels were used to resolve cell lysates and were transferred to nitrocellulose membrane. A total of 2.5% milk in PBST was used to block the membranes at room temperature (RT) for 30 min, and, following that, the membranes were incubated overnight at 4 °C with primary antibodies. The following day, after washing with PBST, the blots were incubated in HRP-conjugated secondary antibodies for 1h at RT. All of the antibodies were diluted in 2.5% milk containing PBST. After 5× washings with PBST, the immunoreactive bands were soaked with enhanced chemiluminescence reagents (ThermoFisher Scientific, Waltham, MA, USA), and then visualized by the BioSpectrum 810 Imaging System (UVP LLC, Upland, CA, USA) and quantified by densitometry using ImageJ software. For immunoprecipitations (IP), cell lysates were prepared in IP-lysis buffer (20 mM Tris-HCl [pH 7.5], 150 mM sodium chloride, 0.5% Nonidet P-40, 1 mM EDTA, and protease inhibitor mixture). The lysates were incubated with 100U of benzonase (70664-10KU; Millipore Sigma, Burlington, MA, USA) at 4 °C for 1 h, followed by mild sonication and centrifugation at 16,000× *g* for 15 min at 4 °C. The resulting lysates were incubated with 1.5–3 µg of specified antibody or control IgG at 4 °C overnight. Next morning, magnetic A/G beads from Pierce (ThermoFisher Scientific) were used to capture the immunocomplexes for 2 h at 4 °C, followed by extensive washing. The immunocomplexes were resolved on SDS-PAGE and analyzed by Western blotting as described above. Antibodies used were: MDM2 (1:1000) (sc-965), p21 (1:1000) (sc-53870), actin (1:3000) (sc-133155), and GST (1:5000) (sc-138) purchased from Santa Cruz Biotechnology (Santa Cruz, CA, USA); HA (1:3000) (W15093A) from BioLegend (San Diego, CA, USA); mouse anti-p53 (1:2000) (DO-1, AHO0152) from Invitrogen (Waltham, MA, USA); rabbit anti-p53 (1:1000) (Cal#BS-8687R) from Bioss Antibodies (Woburn, MA, USA); mouse anti-mouse p53 (1:2000) (1C12, Catl#2524) from Cell Signaling (Danvers, MA, USA).

### 2.4. RNA Isolation and RT-PCR

TRIzol reagent (Invitrogen) was used to harvest total RNA from cells. cDNA was synthesized from 2 μg total RNA using RevertAid First Strand cDNA Synthesis kit according to the manufacturer’s protocol (ThermoFisher Scientific). The levels of various transcripts were measured by qRT-PCR or semiquantitative PCR with primers listed in Supplementary Table S2.

### 2.5. Protein Half-Life Assay

Cells were treated with cycloheximide (CHX), an inhibitor of protein translation, at the indicated times to measure p53 protein stability. The relative level of remaining p53 protein over time was quantified using ImageJ software and normalized using the level of actin. The values for remaining p53 were plotted versus treatment time and the half-life of p53 protein calculated from the graph.

### 2.6. Chromatin Immunoprecipitation (ChIP) Assays

ChIP assays were performed based on previously published protocols [24]. In brief, p53 expression was induced by treating RKO cells with 250 nM camptothecin (CPT) for 16 h, fixed with 1% paraformaldehyde and lysed with lysis buffer (5 mM HEPES pH 8.0, 85 mM KCl, 0.5% NP-40, protease inhibitor cocktail) on ice for 10 min. Nuclei were subsequently collected by centrifugation and lysed in 1× RIPA buffer (50 mM Tris pH 7.4, 150 mM NaCl, 1% NP-40, 0.5% sodium deoxycholate, 0.1% SDS, 2 mM MgCl2, protease inhibitor cocktail) on ice for 10 min. The chromatin was sonicated on ice using Branson 150 Ultrasonics Sonifier (Fisher Scientific, Waltham, MA, USA) at amplitude 43%, 20 s on and 30 s off, for a total of 22 pulses. The chromatin was centrifuged at 16,000× *g* for 15 min at 4 °C, and immunoprecipitated with anti-p53 (DO-1**,** AHO0152, Invitrogen) for ChIP assay. IP with normal mouse IgG was used as a negative control for ChIP. Before setting up IPs, 10% of the lysates were taken out as inputs. The inputs were processed in the same manner as the IP samples. The binding of p53 to the p53-REs in the human *ZFP14* and *p21* promoters was detected by PCR with the primers listed in Supplementary Table S3. The putative p53-REs in the human *ZFP14* gene were identified using a web tool (p53famtag.ba.itb.cnr.it) and also by manual searches in the promoter region 3 kb upstream of TSS and in the entire intron 1 region.

### 2.7. Colony Formation Assays

The protocol for colony formation assays has been described previously [23]. Briefly, 1000 isogenic control or *ZFP14-KO* HCT116 or MCF-7 cells were plated in 3 wells of a 6-well plate and grown until colonies were visible with change in medium every 3 days. Subsequently, the colonies were stained with crystal violet after fixing with methanol: acetic acid (7:1). ColonyArea plug-in tool in ImageJ software was used to quantify the colony area based on the published protocol [25] and represented as percentage of colony area relative to the isogenic control. For colony formations after siRNA treatment, 5 × 10^5^ isogenic control or *ZFP14-KO* HCT116 cells were plated in one well of a 12-well plate followed by transfection with control or *p53* siRNAs. The following day, 1000 cells for each group were plated in one well of a 6-well plate in triplicates and subsequently grown, fixed and quantified as above.

### 2.8. Wound-Healing Assay

HCT116 and MCF7 cells were plated at 50% confluency. Next day, when the cells reached 70% confluency, a scratch was made with a 10 μL pipette tip. Following this, the cells were incubated with fresh medium for 48 h and then captured with Nikon microscope (Nikon Corporation, Tokyo, Japan).

### 2.9. GST Pull-Down Assay

The protocol published by Frangioni and Neel [26] was used to purify GST-ZFP14 wild-type or mutants from bacterial lysates. After purification, 1 μg of glutathione bead bound GST, GST-ZFP14 wild-type or mutants were incubated with lysates from indicated cell lines prepared in lysis buffer (20 mM Tris-HCl [pH 7.5], 150 mM sodium chloride, 0.5% Nonidet P-40, protease inhibitor cocktail) for 4 h at 4 °C and washed extensively using lysis buffer. The pulled-down proteins were resolved by SDS-PAGE, transferred to nitrocellulose membrane and immunoblotted as described above.

### 2.10. Ubiquitination Assay

For in vivo ubiquitination assays, pcDNA3 constructs containing WT-p53 and 2×-Flag-ubiquitin with or without HA-ZFP14 were transfected into HCT116 cells using Jetprime transfection reagent for 20 h, followed by treatment with 20 μM MG132 for 5 h. Following this, cells were lysed in 1× RIPA buffer and the lysates were immunoprecipitated with mouse anti-p53 antibody (AHO0152, Invitrogen) followed by Western blot analysis with HRP conjugated anti-FLAG antibody (1:3000) (Cat#86852, Cell Signaling) or rabbit anti-p53 antibody (1:1000) (Cat#BS-8687R, Bioss Antibodies) to detect poly-ubiquitinated p53.

### 2.11. Statistical Analysis

The graphs for qRT-PCRs and colony formation assays are presented as means ± standard error of the mean (SEM) or means ± standard deviation (SD). Two-tailed Student’s *t*-tests were used to calculate the *p*-values for qRT-PCRs and colony formation assays, and *p*-value < 0.05 was considered statistically significant. *n* = 3 for each experimental data point. Statistical analyses were performed and the graphs were made using Microsoft Excel (Microsoft, Redmond, WA, USA).

## 3. Results

### 3.1. ZFP14 Expression Correlates with p53 in Human Tissues and ZFP14 Is Induced upon DNA Damage in a p53-Dependent Manner

**Figure 1:**
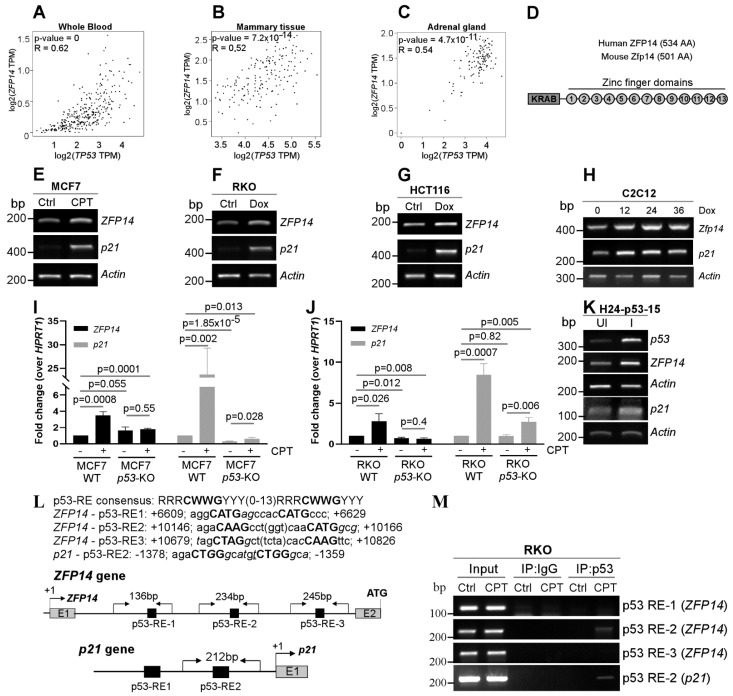
ZFP14 is induced upon DNA damage and is a p53 target. (**A**–**C**) Correlation of *ZFP14* expression with *p53* expression in normal human tissues from GEPIA. (**D**) Schematic showing the various domains and motifs present in human and mouse ZFP14. AA; amino acids. (**E**–**G**) The levels of *ZFP14*, *p21* and *Actin* mRNAs were measured by RT-PCR in MCF7 cells mock-treated or treated with 250 nM CPT (**E**) or RKO cells (**F**) and HCT116 cells (**G**) mock-treated or treated with 250 μg/mL Dox for 18 h. (**H**) The levels of *Zfp14*, *p21* and *Actin* transcripts were measured in mouse C2C12 cells mock-treated or treated with 250 μg/mL Dox for 12-36 h. (**I**,**J**) qRT-PCR was used to measure the levels of *ZFP14* and *p21* mRNAs in MCF7 WT or *p53-KO* cells mock-treated or treated with 250 nM CPT (**I**) and RKO WT or *p53-KO* cells mock-treated or treated 350 μg/mL Dox for 18 h (**J**). (**K**) The levels of *p53*, *ZFP14*, *p21* and *Actin* mRNAs were measured by RT-PCR in H1299 cells (H24-p53-15 clone) uninduced or induced to express p53 for 24 h. (**L**) Sequences of consensus p53-RE, the putative p53-REs located in *ZFP14* intron 1 and p53-RE2 in the p21 promoter. Also shown are the locations of the p53-REs in the human *ZFP14* and human *p21* gene regions and the primer locations used for chromatin immunoprecipitation (ChIP) assays. (**M**) RKO cells were treated with 250 nM CPT for 16 h and the lysates obtained were used to perform ChIP assays using p53 antibody. PCR was performed using primers shown in (**L**) to determine the p53 binding to the *ZFP14* promoter region. p53 binding to the *p21* promoter upon DNA damage was used as a positive control.

KRAB-ZFPs have the unique ability to regulate both p53-mediated transcription and p53 protein stability [12,14,15,16,17,18,19]. In addition, the regulation of KRAB-ZFPs by p53 widens the ability of p53 to indirectly regulate several genes, as KRAB-ZFPs can recognize unique DNA, RNA and protein sequences [2,3,4]. This is important because, out of the several genes identified as p53 targets, only a fraction are directly regulated by p53 and a majority of them are indirect targets [27,28]. Thus, identifying novel KRAB-ZFPs as p53 targets is a key area in cancer research. In order to identify putative KRAB-ZFPs as p53 targets, we examined the co-expression of several KRAB-ZFPs with p53 in normal human tissues using the online tool GEPIA. We found that the expression of *ZFP14* was positively correlated with that of *p53* in several normal human tissue samples (Figure 1A–C and Figure S1A–D). In addition, we also showed the correlation of *ZFP14* expression with that of other p53 target genes, *MDM2* and *CDKN1A* (p21), in normal human tissues (Supplementary Figure S1E–H). Moreover, we examined the correlation of *ZFP14* expression with that of *p53* mRNA expression/mutations in the TCGA pan cancer database. Interestingly, we observed that cancers having a lower *ZFP14* expression were correlated with those having *p53* mutations, indicating that the wild-type *p53* status is required for higher levels of *ZFP14* in cancer patients (Supplementary Figure S1I). ZFP14 (also known as ZNF531) consists of an N-terminal KRAB domain, a linker region and 13 C2H2-type zinc fingers in the C-terminus (Figure 1D). A search for a homolog in mice identified mouse Zfp14 as having an 88% similarity to human ZFP14 (Supplementary Figure S2A). The multi-ZNFs recognize specific DNA, RNA or protein sequences via the variable amino acids present at positions −1, +2, +3 and +6 of each zinc finger domain [2]. As the variable residues for each zinc finger are different, every multi-ZNF protein has a unique zinc finger signature based on the variable residues present in each of its zinc fingers. Because these variable residues decide the substrate identity, we compared the zinc finger signatures of human and mouse ZFP14. Notably, the comparison of the zinc fingerprint between the human and mouse ZFP14 showed those to be identical (Supplementary Figure S2B).

Next, we examined if ZFP14 can be induced upon DNA damage in a p53-dependent manner. For this, we used wild-type p53 containing human cancer cells MCF7, RKO and HCT116, as well as mouse myocyte cells C2C12, and treated them with a DNA-damaging agent doxorubicin (DOX) or camptothecin (CPT). We found that DNA damage led to an increased expression of *ZFP14* along with the positive control *p21* (Figure 1E–H). Importantly, the increase in *ZFP14* expression post DNA-damage was p53-dependent, as we did not observe this increase in *TP53-KO* MCF7 and RKO cells (Figure 1I,J). Moreover, the induction of p53 in H1299 *p53*-null cells also resulted in a robust increase in *ZFP14* expression along with *p21* (Figure 1K). We also confirmed the expression of p53 protein in all of the above samples by Western blotting (Supplementary Figure S3A–H).

To determine if ZFP14 is a direct p53 target, we searched for putative p53-responsive element(s) (p53-RE) in the *ZFP14* genomic locus. We found three putative p53-REs that were all located in the intron 1 of the human *ZFP14* gene (Figure 1L). To determine if any of these putative p53-REs are bound by p53, we performed chromatin immunoprecipitation (ChIP) assays after treating cells with DNA damage. We showed that p53 recognized only p53-RE2, but not p53-RE1 or p53-RE3 (Figure 1M). As a positive control, we showed the binding of p53 to the *p21* promoter following DNA damage (Figure 1M). Thus, ZFP14 is induced by genotoxic stress in a p53-dependent manner.

### 3.2. Human and Mouse ZFP14 Negatively Regulate p53

**Figure 2:**
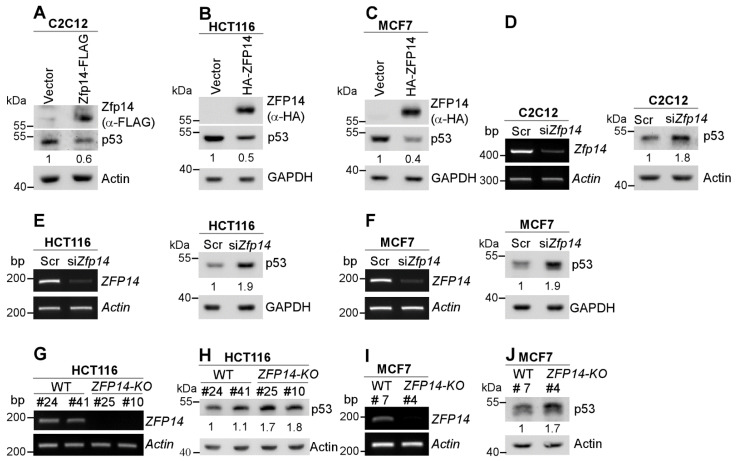
Human and mouse ZFP14 negatively regulate p53. (**A**–**C**) ZFP14, p53 and actin/GAPDH protein levels were measured by Western blotting in mouse C2C12 (**A**), human HCT116 (**B**) and human MCF7 (**C**) cells transiently transfected with either empty vector, mouse Zfp14-FLAG (**A**) or human HA-ZFP14 (**B**,**C**) for 24 h. (**D**–**F**) The levels of *ZFP14* and *actin* mRNAs (left panels) or p53 and GAPDH/actin proteins (right panels) were measured in mouse C2C12 (**D**), human HCT116 (**E**) and human MCF7 (**F**) cells that were transfected with 75 nM scramble or *ZFP14* siRNA for 72 h. (**G**,**I**) The levels of *ZFP14* and *actin* mRNAs were measured in isogenic controls and *ZFP14-KO* HCT116 (clones #25 and #10) cells (**G**) or isogenic control and *ZFP14-KO* MCF7 (clone #4) cells (**I**). (H, J) The levels of p53 and actin proteins were measured in isogenic controls and *ZFP14-KO* HCT116 (clones #25 and #10) cells (**H**) or isogenic control and *ZFP14-KO* MCF7 (clone #4) cells (**J**).

Several genes that are regulated by p53, such as MDM2 and RBM38, in turn regulate p53, forming a feedback loop [29,30]. To test this, we transiently expressed mouse/human ZFP14 in multiple mouse and human cancer cells. We showed that the levels of p53 protein were decreased by the over-expression of mouse Zfp14 in mouse C2C12, as well as by the over-expression of human ZFP14 in HCT116 and MCF7 cells (Figure 2A–C). Conversely, the knockdown of *Zfp14* markedly increased the level of p53 protein in mouse C2C12 cells (Figure 2D). Similarly, the KD of *ZFP14* in HCT116 and MCF7 cells led to a substantial p53 increase (Figure 2E,F). Moreover, we also generated *ZFP14-KO* HCT116 and MCF7 cell lines using CRISPR-Cas9 (Supplementary Figure S4A–C) and tested the p53 levels in those. Examining *ZFP14* mRNA expression in the isogenic controls and *ZFP14-KO* clones revealed no *ZFP14* mRNA in KO clones (Figure 2G,I). Notably, we observed elevated p53 protein in *ZFP14-KO* HCT116 and MCF7 clones compared to isogenic controls (Figure 2H,J). These data indicate that ZFP14 can negatively regulate both mouse and human p53 expression.

### 3.3. ZFP14 Regulates p53 Protein Stability by Associating with MDM2

**Figure 3:**
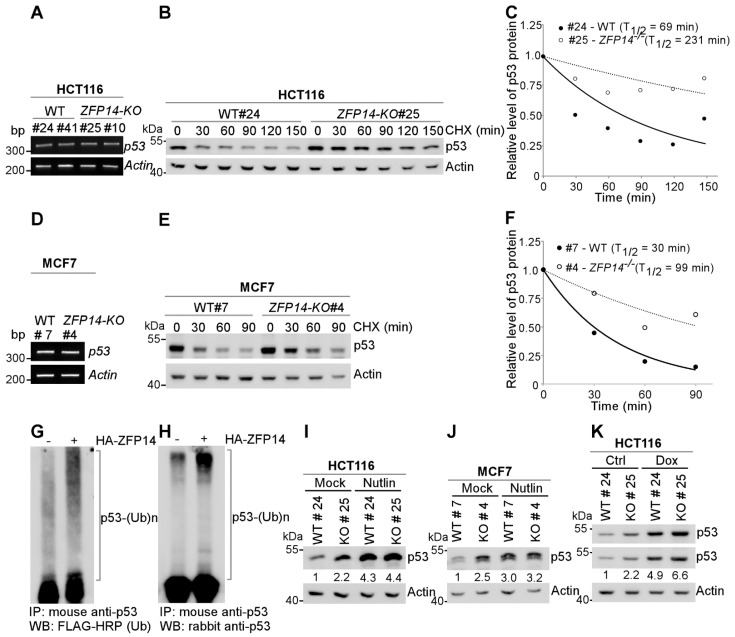
ZFP14 regulates p53 protein stability through MDM2. (**A**,**D**) The levels of *p53* and *actin* mRNAs were measured in isogenic controls and *ZFP14-KO* HCT116 (clones #25 and #10) cells (**A**) or isogenic control and *ZFP14-KO* MCF7 (clone #4) cells (**D**). (**B**,**E**) The levels of p53, vinculin and actin proteins were measured in isogenic control and *ZFP14-KO* HCT116 (clone# 25) cells (**B**) or isogenic control and *ZFP14-KO* MCF7 (clone #4) cells (**E**) mock-treated or treated with cycloheximide (CHX) for indicated times. (**C**,**F**) The relative level of p53 protein in (**B**,**E**) was normalized to actin and the half-life of p53 protein was calculated by plotting the remaining protein over time. (**G**,**H**) pcDNA3-p53 and pcDNA3-2×-Flag-ubiquitin with or without pcDNA3-HA-ZFP14 were transfected in HCT116 cells followed by treatment with 20 µM MG132 for 5 h. Poly-ubiquitinated p53 levels in whole-cell lysates were measured by immunoprecipitation with mouse anti-p53 antibody followed by Western blotting with FLAG-HRP (**G**) or rabbit anti-p53 (H). (**I**,**J**) The levels of p53 and actin proteins were measured in isogenic control and *ZFP14-KO* HCT116 (clone# 25) cells (**I**) or isogenic control and *ZFP14-KO* MCF7 (clone #4) cells (**J**) mock-treated or treated with Nutlin for 16 h. (**K**) The levels of p53 and actin proteins were measured in isogenic control and *ZFP14-KO* HCT116 (clone# 25) cells mock-treated or treated with 250 µg/mL Dox for 18 h.

p53 expression is known to be regulated by two major pathways: transcriptional and post-transcriptional mechanisms [9]. As ZFP14 can act as a transcriptional repressor, we first determined the mRNA levels of *p53* in isogenic control and *ZFP14-KO* cells. We found no difference in the *p53* mRNA levels in *ZFP14-KO* HCT116 and MCF7 cells compared to the isogenic control cells (Figure 3A,D), ruling out the regulation of *p53* mRNA by ZFP14. Next, we looked to determine if ZFP14 can regulate p53 protein stability. For this, we treated isogenic control and *ZFP14-KO* HCT116 and MCF7 clones with cycloheximide (CHX) for various times. We found that *ZFP14* deletion led to an increased p53 protein stability in both HCT116 and MCF7 cells (Figure 3B,E). The p53 protein half-life increased from 69 min in isogenic control HCT116 cells to 231 min in *ZFP14-KO* cells (Figure 3C). Similarly, *ZFP14-KO* MCF7 showed a p53 protein half-life increase to 99 min from 30 min in isogenic control cells (Figure 3F).

As ubiquitination is a major mechanism by which p53 protein stability is regulated, we next investigated whether ZFP14 promotes p53 ubiquitination, resulting in enhanced p53 degradation. For this, we co-expressed p53 and 2×-FLAG-ubiquitin with or without HA-ZFP14 in HCT116 cells followed by treatment with 20 μM MG132 for 5 h. As seen in Figure 3G,H, we observed a robust increase in poly-ubiquitinated p53 upon ectopic expression of ZFP14, suggesting that ZFP14 regulates p53 protein stability by modulating its ubiquitination.

Since MDM2 is the major E3 ubiquitin ligase for p53 that regulates p53 protein stability [31], we next examined if ZFP14 regulates p53 protein stability through MDM2. For this, we examined p53 levels in isogenic control and *ZFP14-KO* clones treated with or without Nutlin-3, an inhibitor of MDM2-p53 interaction. We observed increased p53 protein upon Nutlin treatment in isogenic control cells; however, p53 was not further increased in *ZFP14-KO* HCT116 and MCF7 cells treated with Nutlin (Figure 3I,J), indicating that ZFP14 regulates p53 protein stability through MDM2. In addition, as opposed to the Nutlin treatment above, we showed that the levels of p53 were further increased by genotoxic stress in *ZFP14-KO* cells compared to isogenic control cells (Figure 3K), showing that ZFP14 regulates p53 levels under basal and stress conditions possibly through MDM2.

**Figure 4:**
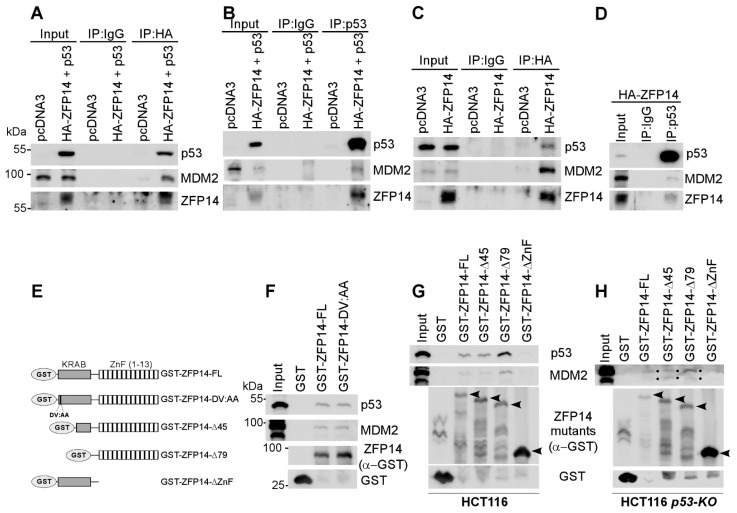
ZFP14 associates with MDM2-p53 complex via its zinc finger domains. (**A**–**D**) Lysates from HCT116 cells transfected with indicated plasmids were subjected to immunoprecipitation using anti-HA (**A**,**C**) or anti-p53 (**B**,**D**) antibodies. The immunoprecipitates were subjected to Western blotting to measure the association of p53, ZFP14 and MDM2 proteins. (**E**) Schematic diagrams and names of all of the GST-fused mutants of ZFP14 used for GST pull-down assays. The DV residues correspond to amino acids 10 and 11, respectively, in human ZFP14. (**F**,**G**) GST pull-down assays were performed by incubating the indicated bacterially purified GST-fusion proteins with HCT116 cell lysates followed by washes and Western blotting using the indicated antibodies. (**H**) GST pull-down assays were performed by incubating the indicated bacterially purified GST-fusion proteins with HCT116-*p53-KO* cell lysates followed by washes and Western blotting using the indicated antibodies.

To determine how ZFP14 regulates p53 protein stability, we examined whether ZFP14 may associate with the p53/MDM2 complex through its interaction with the KRAB-associated protein-1 (KAP1, also called TRIM28) as a part of the MDM2 E3 ubiquitin ligase complex [32]. To test this, we overexpressed p53 and HA-ZFP14 in HCT116 cells and performed co-immunoprecipitation experiments using anti-HA (ZFP14) (Figure 4A) or anti-p53 antibodies (Figure 4B). We found that ZFP14 pulled down both MDM2 and p53 (Figure 4A), indicating that ZFP14 forms a complex with MDM2-p53. Similarly, we showed that p53 was able to pull down both ZFP14 and MDM2 (Figure 4B). Importantly, we tested if ZFP14 can associate with endogenous p53 by performing anti-HA or anti-p53 co-immunoprecipitations in HCT116 cells overexpressing only HA-ZFP14. As seen in Figure 4C, we showed that ZFP14 associated with endogenous p53 and MDM2. Moreover, reciprocal immunoprecipitation with anti-p53 antibodies showed that endogenous p53 was also present in a complex with ZFP14 and MDM2 (Figure 4D).

The N-terminal KRAB domain in KRAB-ZNFs is known to be necessary for their association with MDM2 [24]. Specifically, a conserved aspartate and valine in the KRAB domain has been shown to be essential for the association of the KRAB domain with the KAP1-MDM2 complex [6]. Thus, to test if ZFP14 associates with MDM2 through interacting with KAP1 via the KRAB domain, we mutated the aspartate and valine residues in position 10 and 11 of human ZFP14 to alanine residues (DV:AA mutant) (Figure 4E; also see Supplementary Figure S1A). Next, we performed GST pull-down assays with GST-tagged full-length ZFP14 and the ZFP14 DV:AA mutant using lysates from HCT116 cells. Surprisingly, we found that, like the full-length ZFP14, the DV:AA mutant of ZFP14 was still able to associate with MDM2 and p53 (Figure 4F), indicating that ZFP14 might associate with the MDM2-p53 complex independent of its KRAB domain. To test this theory, we generated various GST-tagged truncated mutants of ZFP14 (Figure 4E). GST pull-down assays using HCT116 cell lysates showed that full-length ZFP14 and ZFP14Δ45, as well as ZFP14-Δ79, were all able to associate with MDM2 and p53 (Figure 4G), consistent with the above findings. Notably, the ZFP14-ΔZnF mutant lacking all of the zinc fingers was defective in bringing down both MDM2 and p53 (Figure 4G), suggesting that, unlike other KRAB-zinc finger proteins, ZFP14 associates with MDM2/p53 via its zinc fingers and possibly enhances MDM2/p53 complex integrity. Further, to examine if ZFP14 can bind to MDM2 independent of p53, we performed GST pull-down assays using HCT116-*p53-KO* cell lysates and showed that the full-length ZFP14 and ZFP14Δ45, as well as ZFP14-Δ79, were all able to bring down MDM2 independent of p53 (Figure 4H).

### 3.4. Decrease in Cancer Cell Growth and Migration upon ZFP14 KO Is p53-Dependent

**Figure 5:**
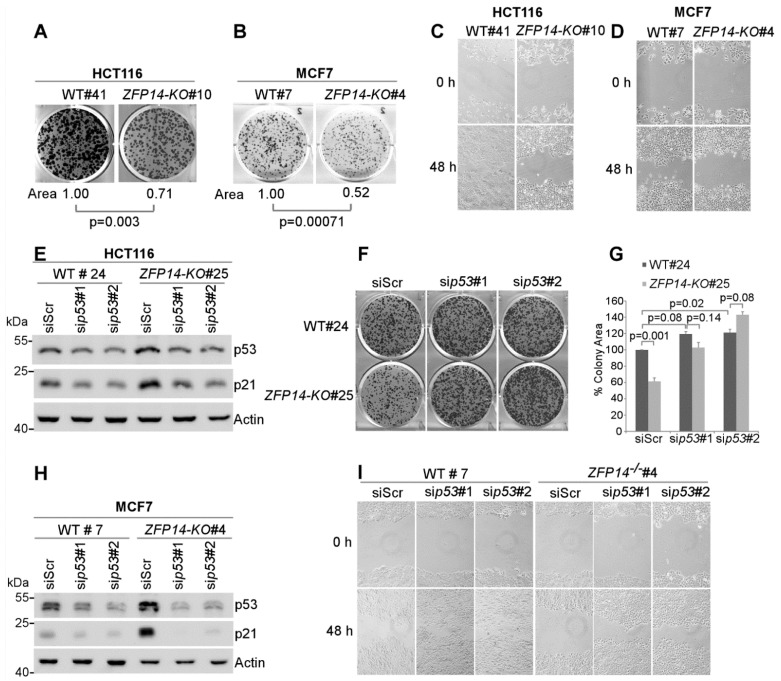
Decrease in cancer cell growth and migration upon *ZFP14 KO* is p53-dependent. (**A**,**B**) Isogenic control and *ZFP14-KO* HCT116 (clone #10) cells: (**A**) cells or isogenic control and *ZFP14-KO* MCF7 (clone #4) cells (**B**) were plated for clonogenic assays and grown for two weeks, followed by fixation and crystal violet staining. ColonyArea plugin in ImageJ was used to quantify the relative colony areas, which are shown below each image. (**C**,**D**) Wound-healing assay was performed with isogenic control and *ZFP14-KO* HCT116 (clone #10) cells (**C**) or isogenic control and *ZFP14-KO* MCF-7 (clone #4) cells (**D**) for a period of 48 h. (**E**) The levels of p53, p21 and actin proteins were measured by Western blotting in isogenic control and *ZFP14-KO* HCT116 cells transfected with scramble, *p53* siRNA#1 or *p53* siRNA#2 for 72 h. (**F**) The cells treated as in (**E**) were plated for clonogenic assays 48 h after transfection and, after two weeks, the colonies were fixed and stained with crystal violet. (**G**) ColonyArea plugin in ImageJ was used to quantify the relative colony areas from (**F**) and plotted as percentage colony area. The area of isogenic control cells transfected with control siRNA was set at 100%. (**H**) The levels of p53, p21 and actin proteins were measured by Western blotting in isogenic control and *ZFP14-KO* MCF7 cells transfected with control, *p53* siRNA#1 or *p53* siRNA#2 for 72 h. (**I**) 72 h after transfection, the cells treated as in (**H**) were subjected to wound-healing assays for 48 h.

As ZFP14 appeared to be a bona fide p53 target that negatively regulated p53, we next assessed the effect of ZFP14 deficiency on cellular oncogenic traits. To test this, we performed clonogenic assays and showed that the ability of *ZFP14-KO* cells to form colonies was significantly decreased compared to isogenic controls in both HCT116 and MCF7 cells (Figure 5A,B). Furthermore, scratch assays showed decreased cell migration in *ZFP14-KO* clones compared to the isogenic control cells in both HCT116 and MCF7 cells (Figure 5C,D), suggesting that ZFP14 promotes cancer cell proliferation and migration.

As the loss of ZFP14 inhibits cell proliferation and migration but increases p53 protein in *ZFP14-KO* cells, we reasoned that the decrease in cell proliferation and migration could be a result of an increased p53. To examine if the reduced cell proliferation upon *ZFP14-KO* is indeed caused by an increased p53, we transiently knocked down *p53* using two different siRNAs in *ZFP14-KO*, as well as in isogenic control HCT116 cells (Figure 5E). We observed decreased p53 levels, as well as its target p21, upon p53 KD (Figure 5E). Next, we used these cells for colony formation assays. Upon transfection with the *p53* siRNAs, isogenic control cells showed a modest increase in the ability to form colonies compared to scrambled siRNA (Figure 5F, top panel and Figure 5G), consistent with the well-known fact that the loss of wild-type p53 promotes cell growth. Notably, the KD of *p53* completely reversed the defect in cell proliferation compared to that by control siRNA in *ZFP14-KO* cells (Figure 5F, lower panel and Figure 5G), indicating that an increased p53 is responsible for decreased cell proliferation in *ZFP14-KO* cells. Similarly, to determine if the decreased cell migration observed in *ZFP14*-deficient cells is also p53-dependent, scratch assays were performed with isogenic control and *ZFP14-KO* MCF7 cells transfected with control siRNA or two separate *p53* siRNAs (Figure 5H). We showed that the KD of *p53* in isogenic control resulted in an increased cell migration, consistent with the well-known fact that the loss of wild-type p53 promotes cell migration. Notably, the decreased migration observed in *ZFP14-KO* cells was ameliorated upon the KD of *p53* (Figure 5I). These data suggest that the decreased tumor cell proliferation and migration observed in *ZFP14-KO* cells is p53-dependent.

## 4. Discussion

KRAB-ZFP genes represent >1% of the genes in humans and other higher vertebrates, with increased numbers correlating with advanced evolution [33]. Thus, they seem to have important functions in mammalian development and diseases, including cancer. Despite their numbers and importance, the function of a majority of them remains ambiguous. Here, we showed a positive correlation of *ZFP14* with *p53* in several normal human tissues and also showed a negative correlation of ZFP14 with p53 mutations in human cancer samples. Subsequently, we identified *ZFP14* as a p53 target gene. Further, we showed that the deletion of *ZFP14* from cancer cells leads to a decrease in their oncogenic properties, which is caused by the negative regulation of p53 by ZFP14.

Several important p53 target genes are still being discovered even after decades of research. Interestingly, a number of zinc finger proteins, such as ZNF365, ZFP871, ZNF750 and ZNF185, have been identified as direct targets of p53 family members [11,12,13,34], indicating the importance of zinc finger proteins in the p53 pathway. This is because zinc finger proteins have the unique ability to bind and regulate DNA and RNA, as well as proteins [2,3,4]. Thus, the p53-mediated regulation of multi-ZFPs increases the repertoire of targets that p53 can modulate indirectly. Moreover, the induction of KRAB-ZFPs could also help in the p53-dependent regulation of endogenous retrolelement and transposon expression that KRAB-ZFPs are known to bind to [33,35]. Here, we found that *ZFP14* expression correlates with that of *p53* in several normal tissues, which prompted us to examine if ZFP14 is transcriptionally regulated by p53. In that respect, we found that ZFP14 is induced by DNA damage in a p53-dependent manner. We also showed that p53 binds to a p53-RE in the intron 1 of the *ZFP14* gene and directly regulates ZFP14 expression.

Multiple p53 target genes, including several zinc finger proteins, in turn negatively or positively regulate p53 expression and/or activity, thus forming regulatory feedback loops [12,30,36]. While some zinc finger proteins, such as ZNF420, PISA and PITA, differentially regulate p53-mediated transcription, protein stability remains the major mechanism controlling p53 function [14,15,37]. In this regard, we and others have shown that ZFP871, ZFP148 and ZER6 regulate p53 protein function by modifying its stability via MDM2, a critical E3 ligase of p53 [12,19,38]. Here, we showed that ZFP14 associates with p53 and MDM2 and negatively regulates p53 protein stability, possibly by enhancing the p53/MDM2 complex integrity. Surprisingly, the GST pull-down assay revealed that ZFP14 associates with p53 and MDM2 through its zinc finger domains but not through its N-terminus KRAB domain, the latter of which is shown to associate with MDM2 via its interaction with KAP1 [32]. Interestingly, other KRAB-ZFPs, such as ZNF420 and ZFP148, also associate with the p53/MDM2 complex through their zinc finger domains [14,19]. Thus, ZFP14 regulates p53 protein stability by associating and possibly enhancing the p53/MDM2 complex integrity. Taken together, we conclude that the p53-MDM2-ZFP14 pathway plays a critical role in tumor cell survival and dissemination.

## 5. Conclusions

The biological functions of most KRAB-ZFPs are unknown and thus the identification of the physiological and cancer-specific function of ZFP14 is significant. Our findings suggest that the mutual regulation of p53 and ZFP14 represents a novel p53-ZFP14 regulatory loop and that ZFP14 plays a role in p53-dependent tumor suppression.

## Data Availability

Not applicable.

## Supplementary Materials

The following supporting information can be downloaded at: https://www.mdpi.com/article/10.3390/cancers14215226/s1, Figure S1: *ZFP14* expression positively correlates with wild-type *p53* in normal human tissues and inversely correlates with mutant p53 in tumor tissues; Figure S2: ZFP14 domain structure, human and mouse ZFP14 similarity and zinc finger signature; Figure S3: p53 expression in various cells induced by DNA damaging agents; Figure S4: Strategy used to knock-out *ZFP14* in cancer cell lines using CRISPR-Cas9; Table S1: siRNA oligonucleotides; Table S2: Primers used for qRT-PCR and RT-PCR; Table S3: Primers used for ChIP-PCR.
