# Supplementary material for: ZFP14 Regulates Cancer Cell Growth and Migration by Modulating p53 Protein Stability as Part of the MDM2 E3 Ubiquitin Ligase Complex
Source: Cancers (Basel). 2022 Oct 25;14(21):5226. doi: 10.3390/cancers14215226 (PMC9655198; doi:10.3390/cancers14215226)
Supplement: Supplementary file 1 [file cancers-14-05226-s001.zip › cancers-1952369-supplementary.pdf]

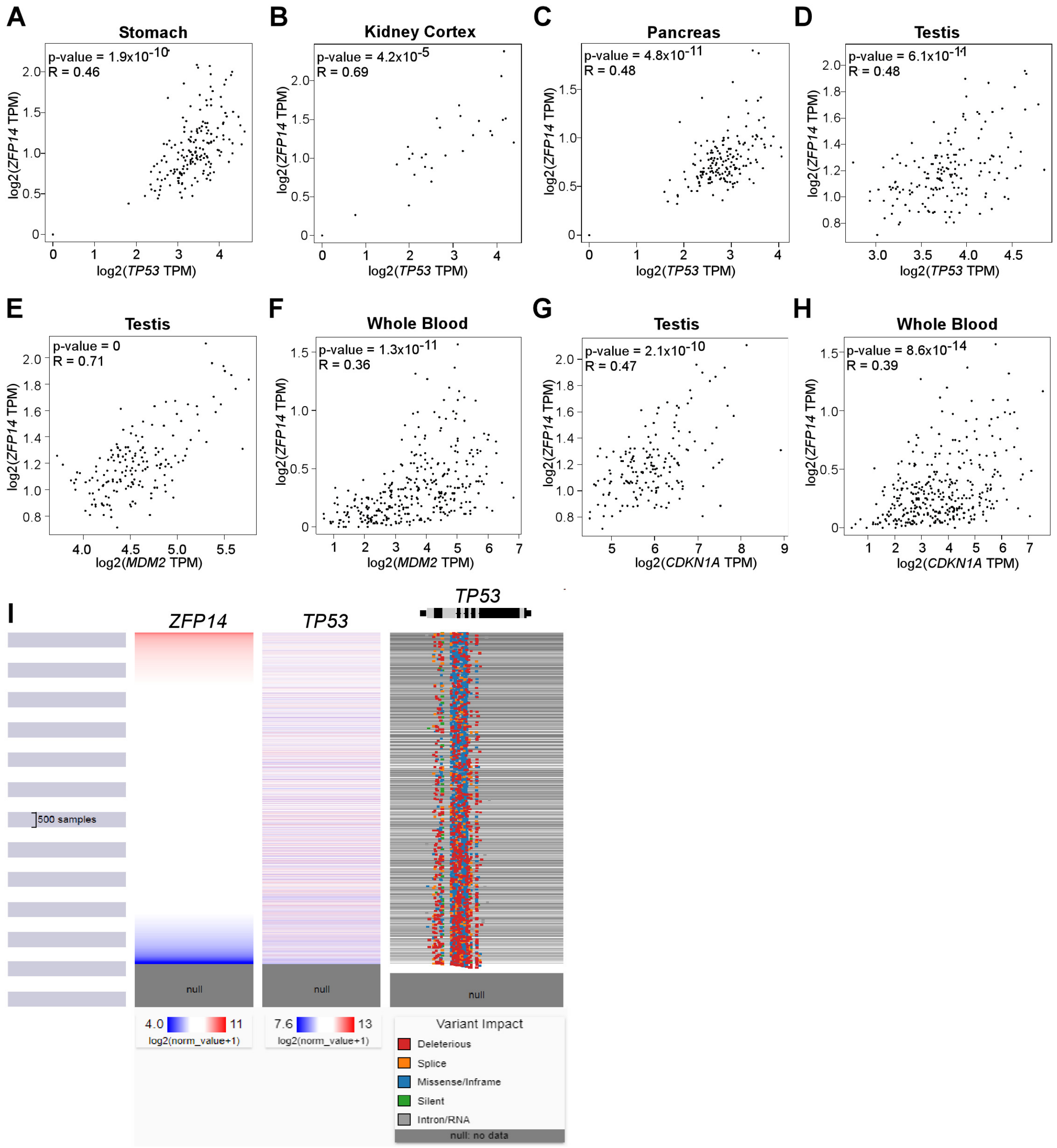

Figure S1. *ZFP14* expression positively correlates with wild-type *p53* in normal human tissues and inversely correlates with mutant *p53* in tumor tissues, related to Figure 1. (**A**–**D**) Correlation of *ZFP14* expression with *p53* expression in normal human tissues from GEPIA. (**E**,**F**) Correlation of *ZFP14* expression with p53 target, *MDM2* in normal human tissues from GEPIA. (**G**,**H**) Correlation of *ZFP14* expression with p53 target, *CDKN1A*, in normal human tissues from GEPIA. (**I**) Correlation of *ZFP14* expression with *p53* mRNA and mutation pattern in human cancer tissues from TCGA pan-cancer database using UCSC Xeno browser.

A

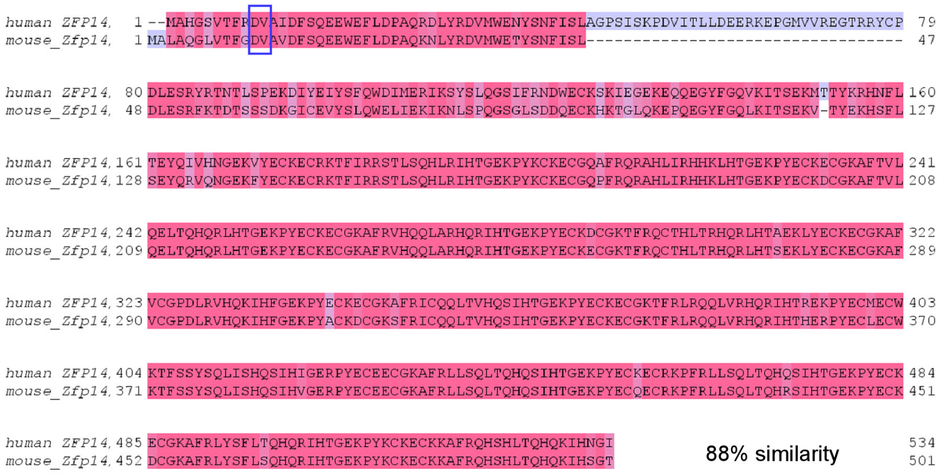

B

All thirteen ZFP14 zinc fingerprints are conserved between human and mouse ZFP14

	1	2	3	4	5	6	7	8	9	10	11	12	13
Human	RSTQ	QAHR	VQEQ	VQQR	QTHR	CPDV	IQQV	LQQR	SSQS	LSQQ	LSQQ	LSFQ	QSHQ
Mouse	RSTQ	QAHR	VQEQ	VQQR	QTHR	CPDV	IQQV	LQQR	SSQS	LSQQ	LSQQ	LSFQ	QSHQ

Figure S2. ZFP14 domain structure, human and mouse ZFP14 similarity and zinc finger signature. (**A**) Sequence comparison between human and mouse ZFP14 proteins. The DV residues highlighted in the box are required for the interaction of KRAB domain with KAP1/TRIM28. The DV residues correspond to amino acids 10/11 in human ZFP14 and 12/13 in mouse ZFP14. (**B**) Comparison of the variable zinc finger signature residues at positions -1, +2, +3 and +6 for each zinc finger domain of human and mouse ZFP14.

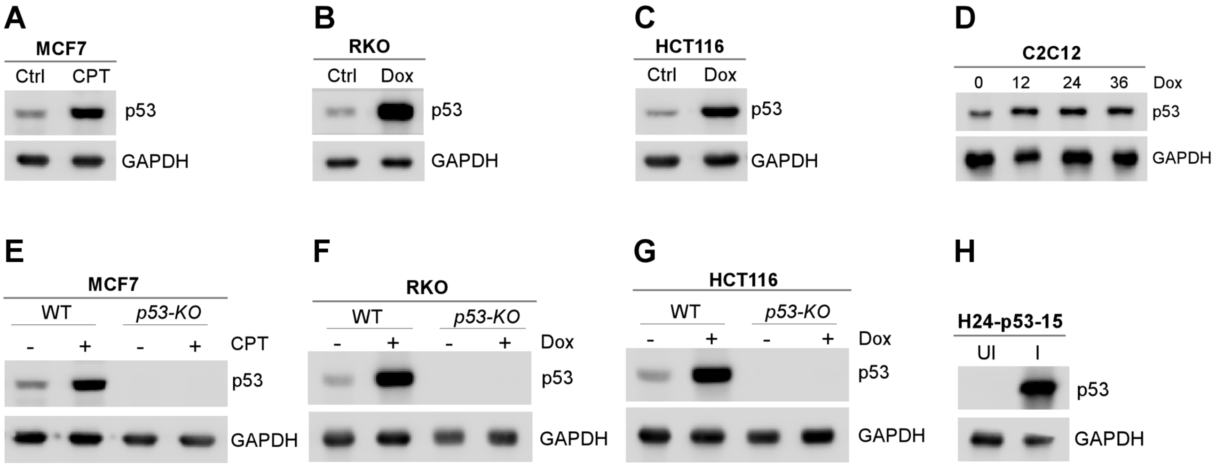

Figure S3. p53 expression in various cells induced by DNA damaging agents, related to Figure 1. (**A–C**) The levels of p53 and GAPDH proteins were measured by western blotting in MCF7 cells mock-treated or treated with 250nM CPT (A) or RKO cells (**B**) and HCT116 cells (**C**) mock-treated or treated with 250μg/ml Dox for 18 h. (**D**) The levels of p53 and GAPDH proteins were measured by western blotting in mouse C2C12 cells mock-treated or treated with 250μg/ml Dox for 12-36 h. (**E–G**) The levels of p53 and GAPDH proteins were measured by western blotting in isogenic control and TP53-KO MCF7 cells mock-treated or treated with 250nM CPT for 18 h (**E**), in isogenic control and TP53-KO RKO cells (**F**) or isogenic control and TP53-KO HCT116 cells (**G**) mock-treated or treated with 250μg/ml Dox for 18 h. (**H**) The levels of p53 and GAPDH proteins were measured by western blotting in H1299 cells (H24-p53-15 clone) uninduced or induced to express p53 for 24 h.

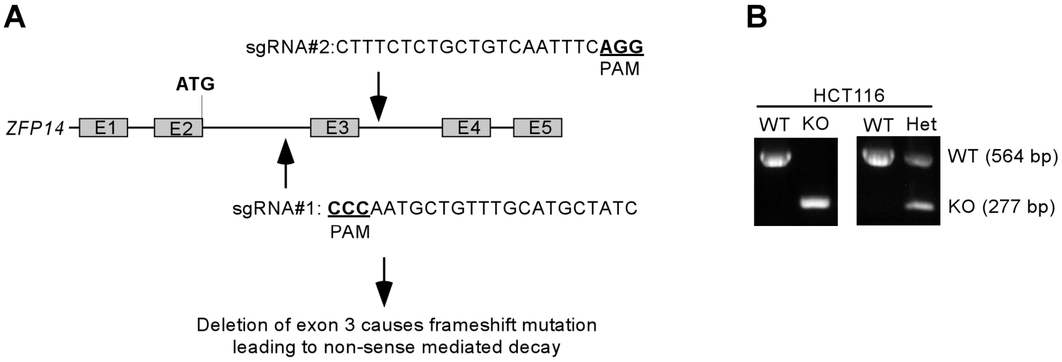

C

```
ZFP14-WT              actggcccctactcatgtcacaagttttcagcccaatgctgtttgcatgctatctgaccc60
HCT116-ZFP14-KO-25    actggcccctactcatgtcacaagttttcagcccaat-----------------------43
HCT116-ZFP14-KO-10    actggcccctactcatgtcacaagttttcagcccaa------------------------36
MCF7-ZFP14-KO-4       actggcccctactcatgtcacaagttttcagcccaa------------------------36
                      ************************************

ZFP14-WT              ctctctgcaatgcaatcctcaatctcccctctttccattttaaaagtatttaatttattt120
HCT116-ZFP14-KO-25    ------------------------------------------------------------43
HCT116-ZFP14-KO-10    ------------------------------------------------------------36
MCF7-ZFP14-KO-4       ------------------------------------------------------------36

ZFP14-WT              tcacaagtaatacctgagtttgttttttcagGGTTCAGTGACATTCAGGGATGTGGCCAT180
HCT116-ZFP14-KO-25    ------------------------------------------------------------43
HCT116-ZFP14-KO-10    ------------------------------------------------------------36
MCF7-ZFP14-KO-4       ------------------------------------------------------------36

ZFP14-WT              AGACTTCTCACAGGAAGAATGGGAATTCCTGGATCCTGCTCAGAGGGACTTATATAGGGA240
HCT116-ZFP14-KO-25    ------------------------------------------------------------43
HCT116-ZFP14-KO-10    ------------------------------------------------------------36
MCF7-ZFP14-KO-4       ------------------------------------------------------------36

ZFP14-WT              TGTAATGTGGGAGAACTACAGCAACTTCATTTCACTAGgtaaggttatttatcccaaatg300
HCT116-ZFP14-KO-25    ------------------------------------------------------------43
HCT116-ZFP14-KO-10    ------------------------------------------------------------36
MCF7-ZFP14-KO-4       ------------------------------------------------------------36

ZFP14-WT              atattagctttctctgctgtcaatttcaggactatatttagaaaaactctctaa354
HCT116-ZFP14-KO-25    -----------------------tttcaggactatatttagaaaaactctctaa68
HCT116-ZFP14-KO-10    -----------------------tttcaggactatatttagaaaaactctctaa67
MCF7-ZFP14-KO-4       -----------------------tttcaggactatatttagaaaaactctctaa67
                                             *******************************
```

Figure S4. Strategy to knock-out *ZFP14* in cancer cell lines using CRISPR-Cas9, related to Figure 2. (**A**) Schematic showing human *ZFP14* gene structure and locations of guides used to delete exon 3 from the *ZFP14* gene. (**B**) Representative image of a PCR gel used to genotype WT, *ZFP14-KO* and *ZFP14-Het* cells. (**C**) The targeted sequence from human *ZFP14* gene is shown along with the deletions in *ZFP14-KO* HCT116 and MCF7 cell lines. Lower case letters indicate part of Intron 2 and Intron 3, whereas upper case letters represent Exon 3. The deleted nucleotides are represented as dash lines; 3-nt PAM sequences are shown in red; the two sgRNA sequences are shown in bold.

Table S1. siRNA oligonucleotides.

Name		Sequence
Scrambled siRNA		5'-GCAGUGUCUCCACGUACUAdTd-3'
si*ZFP14*-human	#1:	5'-CCAACAACUUACUGUUGAUdTdT-3'
	#2:	5'-GGGAAGACUUUUAGAUUAAdTdT-3'
si*ZFP14*-mouse	#1:	5'-CCUUAUCCGACAUCACAAAdTdT-3'
si*p53*-human	#1:	5'-GAAAUUUGCGUGUGGAGUAdTdT-3'
	#2:	5'-GCACAGAGGAAGAGAAUCUdTdT-3'

Table S2. Primers used for qRT-PCR and RT-PCR.

Name	Sequence
Zfp14 (Mouse)	Forward: 5'-TCTTCTGCTTTGGAGCCTCAC-3'<br>Reverse: 5'-CCTGCGGTTCCTTTTGTAGC-3'
Cdkn1a (Mouse)	Forward: 5'-GTACTTCCTCTGCCCTGCTG-3'<br>Reverse: 5'-TCTGCGCTTGGAGTGATAGA-3'
ZFP14 (Human)	Forward: 5'-GGGAATTCCTGGATCCTGCT-3'<br>Reverse: 5'-CTGGACTCCAAATCAGGGCA-3'
ZFP14 (Human)-For KO clones	Forward: 5'-GGAAGGGACAAGAAGATACTGC-3'<br>Reverse: 5'-CCTCAATCTTGCTTTTGCATTC-3'
TP53 (Human)	Forward: 5'-CTCACCATCATCACACTGGAA-3'<br>Reverse: 5'-TCATTCAGCTCTCGGAACATC-3'
CDKN1A (Human)	Forward: 5'-CAGATTTCTACCACTCCAAACGCCG-3'<br>Reverse: 5'-GGACAAGTGGGGAGGAGGAAGTAGC-3'
Actin (Human&mouse)	Forward: 5'-GAAGTACACGAGCCCGGTTT-3'<br>Reverse: 5'-TGGGCTGCGTGGAATTGATG-3'

Table S3. Primers used for ChIP-PCR.

Name	Sequence
ZFP14-RE1 (Human)	Forward: 5'-GAGGCCTCTCCATACTGTTTG-3'<br>Reverse: 5'-CCCTGACACATACAACCTACC-3'
ZFP14-RE2 (Human)	Forward: 5'-CTGTCCCCAGCTTGGATACTAAA-3'<br>Reverse: 5'-TCAAGTGATTATCCTGCCTCAGC-3'
ZFP14-RE3 (Human)	Forward: 5'-TGAGGAGGAGCTGCTCAGAA-3'<br>Reverse: 5'-GAATCGCTTGAACCCAGGAGG-3'
p21-RE2 (Human)	Forward: 5'-GGTCTGCTACTGTGTCCTCC-3'<br>Reverse: 5'-CATCTGAACAGAAATCCCAC-3'
